# KLF7: a new candidate biomarker and therapeutic target for high-grade serous ovarian cancer

**DOI:** 10.1186/s13046-020-01775-9

**Published:** 2020-11-30

**Authors:** Marta De Donato, Gabriele Babini, Simona Mozzetti, Marianna Buttarelli, Alessandra Ciucci, Gloria Arduini, Maria Cristina De Rosa, Giovanni Scambia, Daniela Gallo

**Affiliations:** 1 Unità di Medicina Traslazionale per la Salute della Donna e del Bambino, Dipartimento Scienze della Salute della Donna, del Bambino e di Sanità Pubblica, Fondazione Policlinico Universitario A. Gemelli, IRCCS, Roma, Italy; 2 Dipartimento Universitario Scienze della Vita e Sanità Pubblica – Sezione di Ginecologia ed Ostetricia - Università Cattolica del Sacro Cuore, Largo A. Gemelli 8, 00168 Roma, Italy; 3 Dipartimento Scienze della Salute della Donna, del Bambino e di Sanità Pubblica, Fondazione Policlinico Universitario A. Gemelli, IRCCS, Roma, Italy; 4 Dipartimento di Scienze Biotecnologiche di base, Cliniche Intensivologiche e Perioperatorie, Università Cattolica del Sacro Cuore, Roma, Italy; 5 Istituto di Scienze e Tecnologie Chimiche “Giulio Natta” (SCITEC) – CNR, Roma, Italy

**Keywords:** Ubiquitous Krüppel-like factor, KLF7, Oncogene, Drug target, Ovarian cancer cell lines, Bioinformatics, Virtual screening, Biomarkers, HGSOC, Personalized medicine

## Abstract

**Background:**

In spite of great progress in the surgical and clinical management, until now no significant improvement in overall survival of High-Grade Serous Ovarian Cancer (HGSOC) patients has been achieved. Important aspects for disease control remain unresolved, including unclear pathogenesis, high heterogeneity and relapse resistance after chemotherapy. Therefore, further research on molecular mechanisms involved in cancer progression are needed to find new targets for disease management. The Krüppel-like factors (KLFs) are a family of transcriptional regulators controlling several basic cellular processes, including proliferation, differentiation and migration. They have been shown to play a role in various cancer-relevant processes, in a context-dependent way.

**Methods:**

To investigate a possible role of KLF family members as prognostic biomarkers, we carried out a bioinformatic meta-analysis of ovarian transcriptome datasets in different cohorts of late-stage HGSOC patients. In vitro cellular models of HGSOC were used for functional studies exploring the role of KLF7 in disease development and progression. Finally, molecular modelling and virtual screening were performed to identify putative KLF7 inhibitors.

**Results:**

Bioinformatic analysis highlighted KLF7 as the most significant prognostic gene, among the 17 family members. Univariate and multivariate analyses identified KLF7 as an unfavourable prognostic marker for overall survival in late-stage TCGA-OV and GSE26712 HGSOC cohorts.

Functional in vitro studies demonstrated that KLF7 can play a role as oncogene, driving tumour growth and dissemination. Mechanistic targets of KLF7 included genes involved in epithelial to mesenchymal transition, and in maintaining pluripotency and self-renewal characteristics of cancer stem cells. Finally, in silico analysis provided reliable information for drug-target interaction prediction.

**Conclusions:**

Results from the present study provide the first evidence for an oncogenic role of KLF7 in HGSOC, suggesting it as a promising prognostic marker and therapeutic target.

**Supplementary Information:**

**Supplementary information** accompanies this paper at 10.1186/s13046-020-01775-9.

## Background

Ovarian cancer is the second most common cause of gynaecologic cancer death in women worldwide. Nearly 295,000 women were estimated to have been diagnosed with ovarian cancer and 185,000 to have died from disease in 2018, with rates varying across the world [1]. Over 90% of ovarian malignancies are categorized as epithelial ovarian cancers, and currently five main types are identified: high-grade serous (70%), low-grade serous (< 5%), mucinous (3%), endometrioid (10%), and clear-cell (10%) carcinomas. These are heterogeneous diseases with different epidemiological and genetic risk factors, precursor lesions, patterns of spread, molecular events during oncogenesis, response to chemotherapy, and prognosis [2]. High-Grade Serous Ovarian Cancer (HGSOC) typically presents at advanced stage (III-IV) and, despite the initial response to surgical debulking and first-line therapy with carboplatin and paclitaxel (with or without bevacizumab), most tumours eventually develop drug resistance, with a 5-year survival generally below 30% [3]. Major improvement in maintenance therapy has been seen by incorporating inhibitors against poly (ADP-ribose) polymerase (PARP), but, until now, no significant improvement in overall survival has been achieved in non-BRCA-mutated patients [3, 4]. Starting from this background, there is clearly a need for further understanding the mechanisms behind ovarian cancer progression, this allowing the development of new treatment strategies to overcome drug resistance and improve patient survival.

Kruppel-like family (KLFs) comprises 17 members of zinc-finger transcription factors that recognize the GT/GC box or CACCC element sequences in gene promoter and enhancer regions [5]. KLF family members can be divided into three groups on the basis of functional and structural relationships. Members in group 1 and 3 (KLF3, 8, 12 and KLF9, 10, 11, 14 e 16) act as transcriptional repressor, while members in group 2 serve as transcriptional activators (KLF1, 2, 4, 5, 6 and 7); KLF15 and KLF17 contain no defined protein interaction motifs and are more distantly related [6]. KLFs play critical roles in a multitude of biological processes like proliferation, differentiation, migration, inflammation and pluripotency [5, 7]. Due to their ability to control proliferation in a variety of cell types (through transcriptional control of cell cycle regulatory components) several KLFs have been implicated in the onset and development of cancer. Specifically, there is much evidence demonstrating that expression of these transcription factors is altered in a number of human cancers, where they can act as tumour suppressor or oncogenes, in a context-dependent way [8, 9]. Various recent studies have also identified members of KLF family as novel prognostic biomarkers in cancer [10–12]. With respect to ovarian cancer, only sporadic data are available. Findings come mostly from experimental studies suggesting that KLF2, KLF4, KLF6 and KLF11 act as tumour suppressors in ovarian cancer [13–16] while KLF5, KLF8 and KLF9 might have a potential role in disease development and progression [17–19].

Here, we carried out an extensive meta-analysis of publicly available ovarian cancer transcriptome datasets related to HGSOC patients with advanced stage disease, to explore a possible role of KLF family members as prognostic biomarkers. Univariate and multivariate analyses identified KLF7 as an unfavorable prognostic marker for overall survival in this cohort. In vitro experiments demonstrated that KLF7 is able to promote proliferation, migration, invasion and sphere formation in experimental models representative of HGSOC. Finally, herein we reported results from computational analysis and predictive modeling of discovery of putative small molecule inhibitors of the KLF7-DNA interaction interface.

## Material and methods

### KLF family expression data from publicly available databases

Bioinformatic meta-analyses of transcriptome datasets were performed using the Bioconductor package curatedOvarianData v1.24.0 [20] implemented in R v3.6.1 [21]. The available datasets were filtered in order to keep only samples from patients with serous histology, high-grade and late-stage (FIGO stage III/IV) for which data on age and residual mass after debulking surgery were available. Univariate and multiple Cox-regression analyses (based on gene, age, stage and residual tumor mass) for Overall Survival (OS) were performed on the eligible datasets [i.e. E.MTAB. 386 (https://www.ebi.ac.uk/arrayexpress/experiments/E-MTAB-386/), GSE26712 (https://www.ncbi.nlm.nih.gov/geo/query/acc.cgi?acc=GSE26712), GSE49997 (https://www.ncbi.nlm.nih.gov/geo/query/acc.cgi?acc=GSE49997), GSE9891 (https://www.ncbi.nlm.nih.gov/geo/query/acc.cgi?acc=GSE9891), TCGA-OV RNAseq and TCGA-OV (https://portal.gdc.cancer.gov/)] for each KLF family member. Forest plots, combined hazard ratios with 95% confidence intervals and the corresponding *p*-values were performed for each KLF family member.

Further in depth investigation was performed on two out of the six datasets. In particular, the transcriptome series GSE26712 (with clinical features matched to the Affymetrix human U133A microarray data) was downloaded from the Gene Expression Omnibus (GEO) database [22]. A population cohort of 185 patients with late-stage HGSOC was eligible for the analysis (Table 1). As a second external dataset for KLF7, normalized RNA-Seq results of the TCGA-OV patients’ cohort were downloaded from the National Cancer Institute Genomic Data Commons Data Portal (https://portal.gdc.cancer.gov/) and matched to the corresponding clinical features, as described by the TCGA group [23]. A population cohort of 266 patients was selected as late-stage HGSOC, as indicated in Table 1. The prognostic effect of the various parameters on OS probabilities was estimated using the Kaplan–Meier method and survival curves were evaluated using the log-rank test. The best cutoff for KLF7 expression values was chosen based on the results of Cutoff Finder [24] analyses implemented in R v3.6.1 software environment [21]. Best cutoff value was used to dichotomize patients into low and high KLF7 expression groups. Only variables with *p*-value< 0.1 in the univariate analysis were included in multivariate analysis using the Cox proportional hazards model.

**Table 1:** Clinicopathological characteristics of GSE26712 and TCGA-OV cohorts

Characteristics	GSE26712 No. of patients (%)	TCGA-OV No. of patients (%)
**Histological type**		
HGSOC	185	266
**Median Age, years (range)**	63 (26–84)^a^	59 (31–87)^b^
**FIGO Stage**		
III	146 (78.9)	231 (86.8)
IV	36 (19.5)	35 (13.2)
Not available	3 (1.6)	–
**Residual tumor after primary surgery**		
≤ 1 cm	90 (48.6)	169 (63.5)
> 1 cm	95 (51.4)	69 (25.9)
Not available	–	28 (10.6)

*HGSOC* High-Grade Serous Ovarian Cancer

^a^Patient age was Not Available for three cases

^b^Patient age was Not Available for nine cases

### Cell cultures and reagents

The human ovarian carcinoma cell lines PEO1 and COV318 were obtained from the European Collection of Cell Cultures (ECACC, Salisbury, UK), while NIH:OVCAR-3 and OV-90 from the American Type Culture Collection (ATCC, Milan, Italy). Susan Horwitz (Albert Einstein Medical College) donated the HEY cell line. PEO1 is an adherent cell line derived from a malignant effusion from the peritoneal ascites of a chemotherapy-treated patient with a poorly differentiated serous adenocarcinoma [25]. COV318 is a human ovarian epithelial-serous carcinoma cell line established from a peritoneal ascites [26]. The NIH:OVCAR-3 line was established from the malignant ascites of a chemotherapy-treated patient with progressive adenocarcinoma of the ovary [27]. OV-90 cells were derived from a chemotherapy-naive grade 3, stage IIIC, malignant papillary serous adenocarcinoma [28]. HEY cell line was derived from a human ovarian cancer xenograft originally grown from a peritoneal deposit of a patient with moderately differentiated papillary cystadenocarcinoma of the ovary [29].

PEO1, NIH:OVCAR-3 and HEY were cultured in RPMI 1640 (Roswell Park Memorial Institute Medium) and COV318 in Dulbeccoʼs modified Eagle′s medium (Sigma-Aldrich, St. Louis, MO, USA). OV-90 cells were cultured in 1:1 mixture of MCDB 105 medium (containing a final concentration of 1.5 g/L sodium bicarbonate) and Medium 199 (containing a final concentration of 2.2 g/L sodium bicarbonate). Medium was supplemented with 10% fetal bovine serum (FBS) for PEO1, COV318, NIH:OVCAR-3 and HEY and 15% FBS for OV-90, plus MEM (Minimum Essential Medium) Non-Essential Amino Acid 1%, glutamine 1 mM and kanamycin 1%. Sodium pyruvate 2 mM was also added to PEO1 medium (Sigma-Aldrich). Cells were grown in a fully humidified atmosphere of 5% CO_2_/95% air, at 37 °C. Cells were routinely tested for mycoplasma (MycoAlert mycoplasma detection kit, LONZA, Rockland, ME, USA) and validated by STR (Short Tandem Repeat) DNA profiling (BMR Genomics srl, Padua, Italy).

### KLF7 silencing

Silencing of KLF7 gene expression in OV-90 and PEO1 cells was obtained by transfection with Transfectin (Bio-Rad, CA, USA) and specific siRNAs (siKLF7) (ON-TARGETplus SMARTpool siRNA KLF7, Dharmacon, Lafayette, CO), while a non-targeting siRNA pool was used as control (siC) (ON-TARGETplus Non-targeting Control Pool, Dharmacon, Lafayette, CO). Sequence information related to ON-TARGETplus SMARTpool siRNA KLF7 (pool of 4 siRNA) are provided in Supplementary File 1 (Table S1A). Silencing efficiency was verified by RT-qPCR using primers reported in Table S1B. Cells transiently transfected with siKLF7 or siC were used to perform in vitro assays.

### Proliferation assay

For proliferation assay, 1.2 × 10^6^ OV-90 cells and 1.5 × 10^6^ PEO1 cells were seeded in corning flask (25 cm^2^) in complete culture medium and transfected with siKLF7 or siC. After 24 h, 48 h and 72 h from transfection, cells were harvested by trypsinization and viable cells counted with NucleoCounter NC-200 (Chemometec, Lillerød, Denmark). The mean cell proliferation at Time x (Tx) was expressed as average percentage increase relative to T = 0 h (T0). Experiments were performed at least three times.

### Transwell migration and invasion assays

For both Transwell migration and invasion assays, siC and siKLF7 OV-90 (10,000 cells) and PEO1 (100,000 cells) were added into the upper chamber of the insert (8 μm pore size; Corning, NY, USA) after 24 h from transfection and further 24 h of serum starvation. For invasion assays, the upper chamber of the insert was precoated with Matrigel (Corning, NY, USA). In both assays, the lower chamber contained medium with 10% FBS as chemoattractant. Cells were incubated for 24 h at 37° in a 5% CO_2_ atmosphere, inserts washed with PBS (Phosphate-buffered saline) and cells on the top surface of the insert removed with a cotton swab. Cells adhering to the lower surface were fixed with ethanol, stained with crystal violet, washed with H_2_O and counted under a microscope. Experiments were performed at least three times. The relative migrated or invaded cell numbers were expressed as the percentage of controls.

### Real-time quantitative PCR

Real-time qPCR on mRNAs was performed as previously described [30] using the primers listed in Supplementary File 1 (Table S2). The geometric mean of SNRPD3 and RPLP0 was taken as reference genes, following GeNorm algorithm [31]; relative quantification of target mRNA was performed according to the ΔΔCt method [32]. Experiments were performed at least three times.

### Western blotting

Western blot analysis of total cell lysates (30 μg) was performed as previously described [30], using the following antibodies: anti-KLF7 (sc-101,034, Santa Cruz, Santa Cruz, CA, USA, 1:1000); anti-SNAIL (#3879, Cell Signaling Technology Inc., Danvers, MA, 1:1000); anti-ZEB2 (ab25837, Abcam, Cambridge, UK, 1:1000); anti-VIM monoclonal antibody (sc-73,259, Santa Cruz, 1:1000); anti-CD44 (M7082, Dako Agilent Technologies, Santa Clara, CA, USA, 1:500); anti-GAPDH (ab8245, Abcam, 1:5000). Blots were visualized by enhanced chemiluminescence system (Amersham Biosciences, Buckinghamshire, UK) using a Chemidoc imaging system (Bio-Rad). Experiments were performed at least three times. Proteins were densitometrically quantified using Imager ChemiDoc™ XRS+ Software (Bio-Rad, Version 6.0.1.34). All proteins were normalized to GAPDH loading control.

### Zymography

Activities of matrix metalloproteinases 2 and 9 (MMP2 and MMP9) in siC and siKFL7 OV-90 and PEO1 cells were determined by gelatin zymography. Forty-eight hours after transfection, cell culture media was changed to serum-free media and cells were incubated for another 24 h. Culture media were mixed with sample buffer and loaded for SDS-PAGE. Gelatinase zymography was performed in 10% SDS polyacrylamide gel in the presence of 0.1% gelatin under non-reducing conditions. Following electrophoresis the gels were washed three times in 2.5% Triton X-100 for 20 min at room temperature to remove SDS. The gels were then incubated at 37 °C overnight in substrate buffer containing 50 mM Tris-HCl and 5 mM CaCl_2_, 0.15 M NaCl and 1 μM ZnCl_2_ and stained with 0.5% Coomassie Blue R250 in 50% methanol and 10% glacial acetic acid for 30 min and destained. Experiments were performed at least three times.

### Fluorescence microscopy

Twenty-four hours after transfection, siC and siKFL7 OV-90 and PEO1 cells were seeded in 4-well chamber slides in complete growth medium and incubated for further 48hs. Thereafter, cells were fixed in 4% paraformaldehyde for 20 min at 20 °C and then blocked with 5% v/v goat serum in PBS for 1 h. Immunofluorescence staining was obtained using anti-CD44 (M7082, Dako Agilent Technologies, 1:200), following overnight incubation at + 4 °C. After washing, cells were incubated with secondary antibody anti-mouse Alexa Fluor-488 conjugate (1:200) (Thermo Fisher Scientific, Walthman, MA, USA), in the dark for 30 min at 20 °C. Coverslip was mounted onto slides using an antifade mounting reagent containing DAPI. Slides were observed under a fluorescence microscope (Leica Biosystems, Newcastle, UK) using a 40X objective. Appropriate controls were included to evaluate non-specific staining of the secondary antibody or background autofluorescence.

### Spheroid cultures

For spheroid cultures, cells were mixed with a solution of VitroGel 3D-RGD (TheWell, Bioscence, NJ, USA)/0.5X PBS at a 3:1 ratio to obtain the final cell concentration of 2 × 10^5^ cells/mL. Hydrogel/cell mixture was added to well plate and then incubated at room temperature for 20 min. After stabilization, complete cell culture medium was added on the top of the hydrogel. The 3D cell cultures were maintained for 10 days and continuously monitored using a microscope DM IL LED (Leica Microsystems). Spheroids were first recovered from hydrogel by VitroGel Cell Recovery Solution (TheWell, Bioscence), according to the manufacturer’s protocol, and then measured (diameters, μm), using Leica Application Suite (LAS) analysis. At least 20 spheroids were analyzed for each data point. Experiments were performed at least three times. For immunofluorescence analysis, spheroids were fixed in 4% paraformaldehyde for 20 min at room temperature, and permeabilized in 0.5% v/v Triton X-100 in PBS for 10 min, prior to be blocked with 20% v/v serum and 0.1% v/v Triton X-100 in PBS for 1 h. Immunofluorescence staining was obtained using anti-VIM (Clone V9, Ready-to-Use, Dako, Agilent Technologies, Santa Clara, CA), following overnight incubation at 4 °C. After washing, cells were incubated with secondary antibody anti-mouse Alexa Fluor-488 conjugate (Thermo Fisher Scientific) and DAPI in the dark for 30 min and 5 min, respectively, at room temperature. Spheroids were recovered from hydrogel by VitroGel Cell Recovery Solution and mounted onto slides that were observed under a fluorescence microscope (Leica Biosystems, Newcastle, UK) using a 100X oil immersion objective. Appropriate controls were included to evaluate non-specific staining of the secondary antibody or background autofluorescence.

### Molecular modelling

Homology modeling was performed using Discovery Studio v.19 (Dassault Systems) software suite. The Kruppel-like factor 7 (KLF7) isoform 1 sequence was subjected to BLAST search (https://blast.ncbi.nlm.nih.gov/Blast.cgi) for template identification. Due to the low alignment coverage (32–28% for the top template structures) we modelled only the three zinc-fingers, encompassing residues 218–302. Clustal Omega (https://www.ebi.ac.uk/Tools/msa/clustalo) was used for sequence alignment with the template structure, identified in the crystal structure of the zinc finger domain of KLF4 bound to its target DNA (PDB: 2WBS, 76.47% sequence identity [33]. The alignment was then submitted to the program Modeller [34] as implemented in Discovery Studio to generate 20 models of the zinc-finger domain of the KLF structure bound to the DNA, using a procedure we already applied [35]. The models were optimized by a short simulated annealing refinement protocol, and their consistency was evaluated on the basis of the probability density function (PDF) violations provided by the program. During homology modelling the three zinc-fingers atoms were constrained to the template coordinates. The generated structure was validated using Procheck [36], Verify3D [37] and Prosa-Web [38].

### Virtual screening procedure

The homology model of KLF7 was prepared for docking using the Protein Preparation Wizard of Maestro (Schrödinger LLC, Release 2017–4, New York, NY, 2017). The imported structure was submitted to the protein preparation process which included correcting mislabeled elements, adding hydrogen atoms, assigning bond orders and performing restrained energy minimization using the OPLS_2005 force field [39]. To assess the druggability of KLF7, the site recognition software SiteMap (Maestro, Schrödinger, LLC) was run on the model structure after removal of DNA. The algorithm located potential binding sites evaluating cavity size, exposure to solvent, hydrophobic/hydrophilic balance, and hydrogen bonding. The settings used involved the generation of at least 15 site points per reported site. The SiteScore determines the druggability of the selected pocket and the threshold value for recognition as a drug-binding site is 0.80. The binding sites with highest Sitescore were considered for docking studies. For comparison, the binding pockets of KLF7 were also predicted from the metaPocket server (http://projects.biotec.tu-dresden.de/metapocket) [40]. The NCI Diversity Set III (2243 molecules) from ZINC database [41] and the Maybridge HitFinder collection (14,400 compounds representing the drug-like diversity) were used as libraries for the virtual screening experiment. Specific filters based on drug-like properties [42] and elimination of pan-assay interference compounds (PAINS) [43] were applied. The final database of small molecules (13,552 compounds) was prepared for docking using the LigPrep tool (Schrödinger 2017–4) which generates a minimized conformation of each ligand, and assigns likely protonation states at pH 7 ± 2 and tautomers. All ligands were docked into the druggable site identified by SiteMap and metaPocket using inner and outer receptor grid boxes of 10 Å and 16 Å, respectively. Flexible ligand docking was performed using the Glide program [44] (Schrödinger Release 2017–4) and employing the standard precision mode (SP) followed by the extra precision mode (XP) which uses a more optimized scoring function and a more extensive search of ligand conformations. The MM-GBSA rescoring was performed on the 50% top compounds from XP docking and was used to select virtual hits.

### Statistical analysis

In vitro data were analyzed for homogeneity of variance using an F test. If the variances were heterogeneous, log or reciprocal transformations were made in an attempt to stabilize the variances, followed by Student’s t-test. If the variances remained heterogeneous, a non-parametric test such as the Mann–Whitney U test was used. *P* values were two-sided, with *p* < 0.05 considered as significant. Statistical Analysis was performed using GraphPad Prism 6 (GraphPad Software, Inc. La Jolla, CA, USA) and StatPlus Version v6 (AnalystSoft Inc., Walnut, CA).

## Results

### KLF7 is an independent prognostic factor for OS

**Fig. 1:**
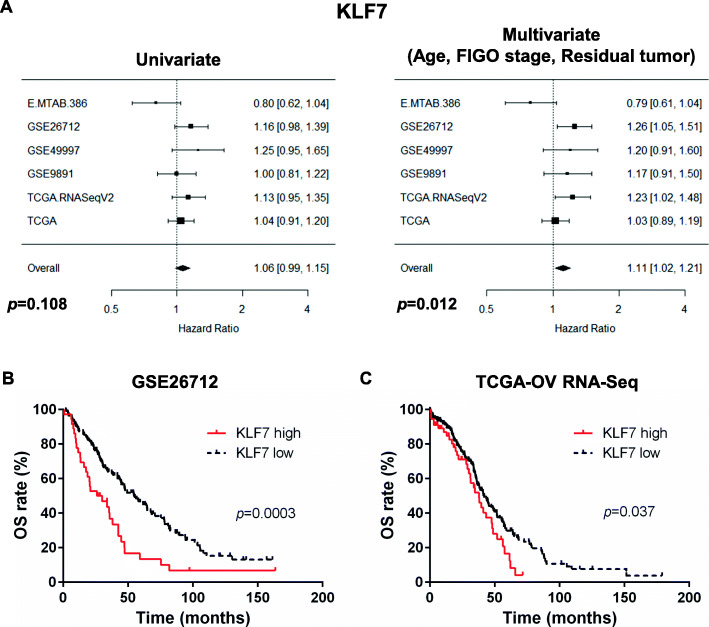
KLF7 is a powerful prognostic biomarker for Overall Survival (OS) in late-stage HGSOC patients. **a** The prognostic value of KLF7 was explored in six publicly available ovarian cancer transcriptome datasets. **b, c** Kaplan-Meier survival curves for the probability of OS in GSE26712 patients cohort (**b**, *n* = 185) and TCGA-OV patients cohort (**c**, *n* = 266). KLF7 expression values were converted into discrete variables by dividing the available population cohorts into “high KLF7” and “low KLF7” based on the cutoff suggested by CutOff Finder: **b** KLF7 Cutoff = 6.861; **c** KLF7 Cutoff = 175. *P*-value in the plot represents the result of log-rank test

Bioinformatic meta-analysis of six publicly available ovarian cancer transcriptome datasets highlighted KLF7 as the most significant prognostic gene, among the 17 family members, after multiple Cox-regression models corrected for age, FIGO stage and residual tumor after debulking surgery. Five out of six datasets showed the same trend indicating KLF7 as unfavorable prognostic biomarker (Overall HR: 1.11, 95% CI: 1.02–1.21, *p* = 0.012), as shown in Fig. 1a. KLF2 and KLF5 resulted as slightly less significant prognostic factors after multivariate Cox-regression models (Supplementary File 2). Among these potentially clinically relevant biomarkers, we focused on the role of KLF7 in the genesis and development of HGSOC, due to the lack of prior research on this topic and the potential for target druggability because of its overexpression in the disease. Results for the remaining KLF family members are available as Supplementary File 2.

The prognostic value of KLF7 expression in HGSOC was further evaluated in two of these large public clinical database which integrate gene expression data with patient survival. Specifically, the datasets chosen were the biggest cohort (after TCGA-OV) of late-stage HGSOC analyzed with a microarray technology (i.e. GSE26712) and the only RNA-seq dataset available (i.e. TCGA-OV). Overall Survival analyses of these two publically available datasets of late-stage HGSOC were performed with the aid of CutOff Finder [24] to identify the best potential cutoff for categorizing the cohorts into “high” or “low” KLF7 expression. Regarding the GSE26712 dataset, results obtained in univariate log-rank test showed that low levels of KLF7 are associated to a longer median overall survival (low KLF7: 55.2 Months vs high KLF7: 28.1 Months, HR = 2.6, 95% CI = 1.6–4.4, *p* = 0.0003) (Fig. 1b, Table 2). In multivariate Cox regression analysis, after adjusting for age and residual tumor after primary surgery, KLF7 was identified as a powerful predictor of OS (*p* < 0.0001, Table 2).

**Table 2:** Univariate and Multivariate analysis of factors affecting OS in GSE26712 cohort

Outcome and variables	Univariate		Multivariate	
	HR (95%CI)	***P***	HR (95%CI)	P*
**Age (yrs)**				
≤ 63				
> 63	1.6 (1.1–2.3)	0.007	1.6 (1.1–2.2)	0.01
**FIGO Stage**				
III				
IV	1.4 (0.9–2.3)	0.1	–	–
**Residual tumor**				
≤ 1 cm				
> 1 cm	1.7 (1.2–2.4)	0.003	1.6 (1.1–2.3)	0.009
**KFL7**				
Low expression				
High expression	2.6 (1.6–4.4)	0.0003	2.2 (1.4–3.4)	< 0.0001

*OS* Overall Survival, *HR* Hazard Ratio, *CI* Confidence Interval

*P values were derived from the Cox proportional hazards model. Only variables with *p*-value < 0.1 in the univariate analysis were included in multivariate model. χ^2^ of the model = 28.15, *p*-value < 0.0001

For TCGA-OV analysis results obtained in univariate log-rank test showed that low levels of KLF7 are associated to a longer median overall survival (low KLF7: 41.9 Months vs high KLF7: 37.8 Months, HR = 1.6, 95%CI = 1.0–2.4, *p* = 0.037) (Fig. 1c, Table 3). In multivariate Cox regression analysis, after adjusting for age, KLF7 was identified as an independent predictor of OS (*p* = 0.03, Table 3).

**Table 3:** Univariate and Multivariate analysis of factors affecting OS in TCGA-OV cohort

Outcome and variables	Univariate		Multivariate	
	HR (95% CI)	***P***	HR (95% CI)	P*
**Age (yrs)**				
≤ 59				
> 59	1.3 (1.0–1.8)	0.09	1.4 (1.0–1.9)	0.06
**FIGO Stage**				
III				
IV	1.1 (0.7–1.8)	0.7	–	–
**Residual tumor**				
≤ 1 cm				
> 1 cm	1.1 (0.8–1.6)	0.5	–	–
**KFL7**				
Low expression				
High expression	1.6 (1.0–2.4)	0.037	1.5 (1.0–2.3)	0.03

*OS* Overall Survival, *HR* Hazard Ratio, *CI* Confidence Interval

*P values were derived from the Cox proportional hazards model. Only variables with *p*-value < 0.1 in the univariate analysis were included in multivariate model. χ^2^ of the model = 7.31, *p*-value = 0.0258

### KLF7 expression in HGSOC cell lines

**Fig. 2:**
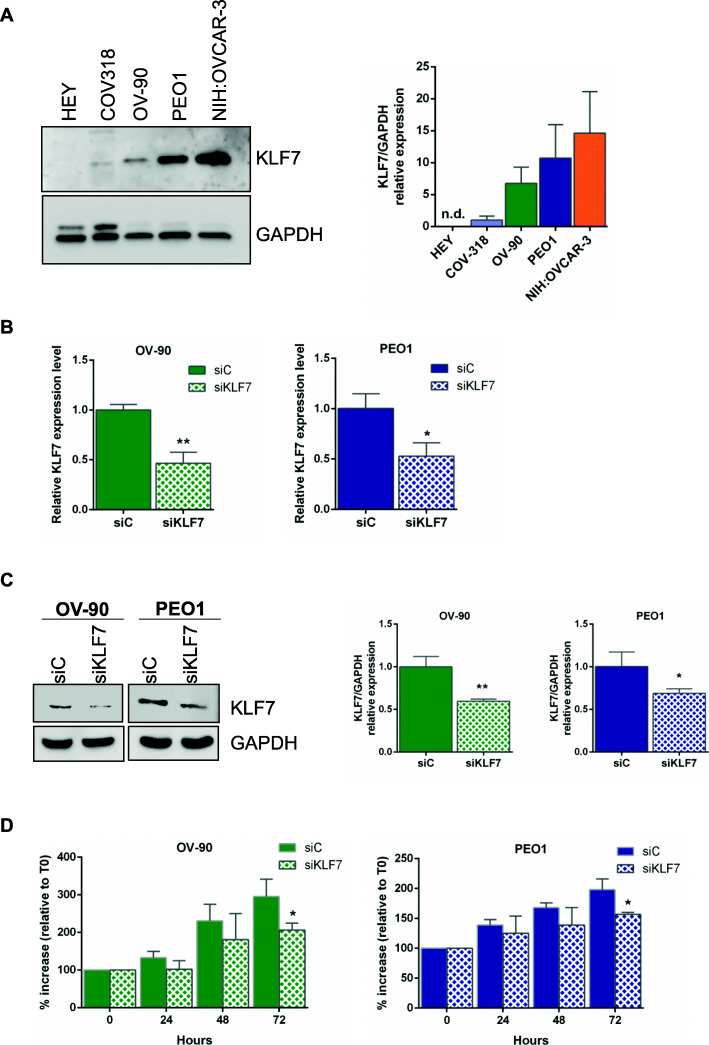
KLF7 silencing inhibits proliferation of high-grade serous ovarian cancer (HGSOC) cells. **a** Representative western blot and densitometric analysis of KLF7 protein expression in a panel of HGSOC cell lines. GAPDH was used as loading control. **b** Relative KLF7 mRNA expression assessed by RT-qPCR analysis in OV-90 and PEO1 cells transiently transfected with specific siRNAs (siKLF7) and non-targeting siRNA pool as control (siC). Data are presented as fold change, calculated with the ΔΔCt method, using siC as reference sample. **c** Representative western blot and densitometric analysis of KLF7 protein expression in OV-90 and PEO1 cells transiently transfected with siKLF7 and with siC, as control. Cells were harvested 72 h post transfection and cell lysates were subjected to western blot with the specific antibody. GAPDH was used as loading control. **d** Bar chart representing proliferation assay for transfected OV-90 and PEO1 cells compared to respective control cells. Viable cells were counted at 24 h, 48 and 72 h from transfection. The mean cell proliferation at Time x (Tx) was expressed as average percentage increase relative to T = 0 h (T0). For all experiments, bars and error bars refer to mean and SD (Standard Deviation) of three experiments. N.d. = not detectable. To establish statistically significant differences, unpaired t-test was carried out. *p < 0.05; **p < 0.01

As a first step in the investigation of the role of KLF7 in cancer development, we assessed protein expression in a panel of cell lines selected among those considered as really representative of HGSOC [45–47]. Western blot analysis revealed different expression levels of KLF7 in the HGSOC cell panel (Fig. 2a).

### KLF7 knockdown inhibits proliferation of HGSOC cells

We used transiently silenced OV-90 and PEO1 cell lines to investigate KLF7 role in cell proliferation. The NIH-OVCAR3 cell line was not included in the study, based on the consideration that the high KLF7 protein levels in this cellular model, along with the observed protein stability in our experimental conditions, would have made it difficult to modulate protein levels in silencing experiments.

A downregulation at RNA and protein level was evident at 72 h post transfection for both cellular models (Fig. 2b and c). Silencing of KLF7 resulted in significant inhibition of cell growth at 72 h for both cell lines compared with scrambled siRNA cells (Fig. 2d, *p* < 0.05). These results indicate that the higher expression of KLF7 is closely associated to the proliferation of HGSOC cells.

### KLF7 knockdown inhibits migration and invasion of HGSOC cells

**Fig. 3:**
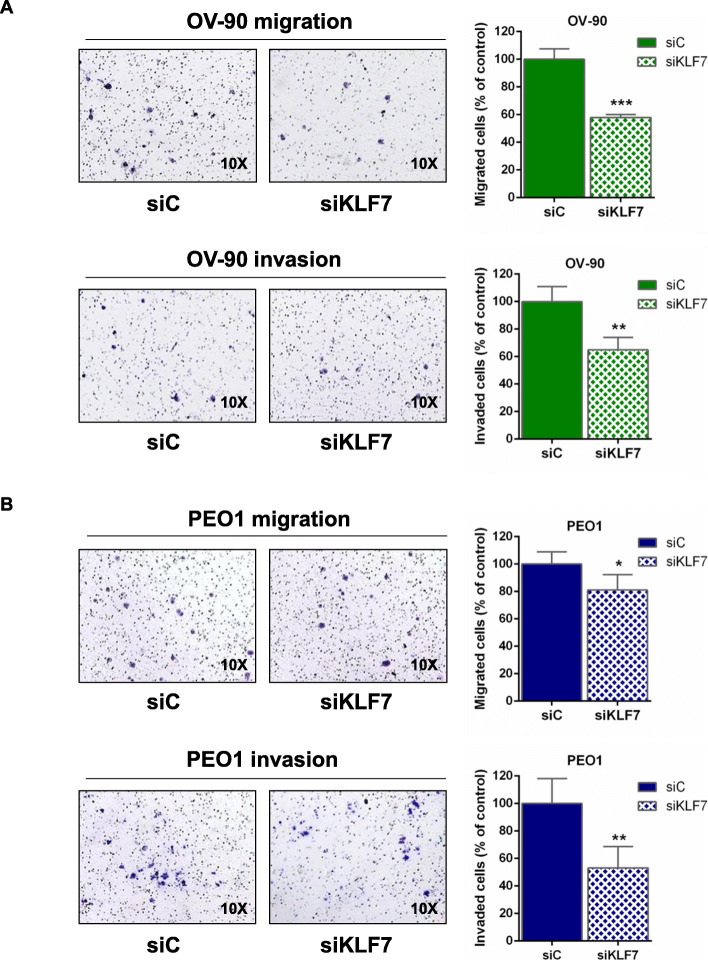
KLF7 silencing inhibits cell migration and invasion of HGSOC cells. Transwell migration and invasion assays in (**a**) OV-90 and (**b**) PEO1 cells transiently transfected with siKLF7 and with siC, as control, and representative picture (left panels). Values are expressed as percentage of migrated or invaded cells relative to control cells. Bars and error bars refer to mean and SD (Standard Deviation) of four experiments. To establish statistically significant differences, unpaired t-test was carried out: *p < 0.05; **p < 0.01 and ***p < 0.001

We performed a transwell migration assay in OV-90 and PEO1 cells after gene silencing to investigate KLF7 role in regulating cell migration. Successful silencing was confirmed (data not shown) and results demonstrated that KLF7 depletion caused a strong reduction in cell migration in OV-90 (*p* < 0.001) and a minor, albeit significant reduction in PEO1 cells, compared to control (*p* < 0.05) (Fig. 3a and b). Analysis of cell invasion corroborated these data, showing a reduced invasion of KLF7-silenced cells compared to control (*p* < 0.01 for both OV-90 and PEO1, Fig. 3a and b). These results indicate that KLF7 is involved in regulating HGSOC cell motility and invasion.

### KLF7 knockdown suppresses expression of EMT-inducer genes

The epithelial–mesenchymal transition (EMT) is mainly regulated by a small number of transcription factors (TFs). They belong to the Snail (SNAIL, SLUG), basic helix-loop-helix (TWIST1 TWIST2), and ZEB (ZEB1, ZEB2) families [48, 49]. Individual EMT-TFs regulate in turn the expression of common, but also specific, target genes, which they can either repress or activate [48, 49]. In order to study the molecular basis underlying the biological effects elicited by KLF7 silencing, the mRNA and protein expression of EMT-TFs, along with their downstream target genes, E-cadherin (CDH1), Vimentin, and matrix metalloproteinases 2 and 9 (MMP2 and MMP9) were analysed in OV-90 and PEO1 cells after transfection with siKLF7 or siC.

**Fig. 4:**
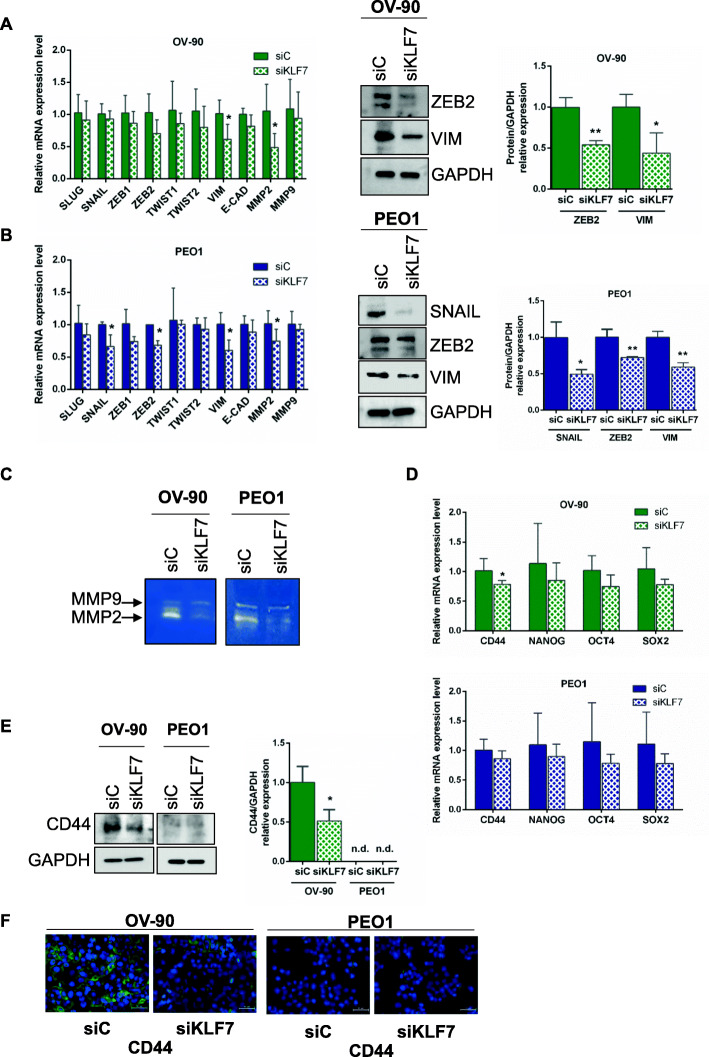
KLF7 silencing modulates epithelial-mesenchymal transition (EMT) and ovarian cancer stem cell markers in HGSOC cells. **a, b** Relative EMT markers expression assessed by RT-qPCR analysis, 72 h post transfection, in OV-90 (**a**) and PEO1 (**b**) cells transiently transfected with specific siRNAs (siKLF7) and non-targeting siRNA pool as control (siC). Representative western blot and densitometric analysis of EMT markers expression in OV-90 (**a**) and PEO1 (**b**) cells transiently transfected with siKLF7 and with siC, as control. Cells were harvested 72 h post transfection and cell lysates were subjected to western blot with specific antibodies. GAPDH was used as loading control. **c** Gelatin zymography: activity of MMP2 and MMP9 was assessed in serum-free cell culture media of OV-90 and PEO1 cells transiently transfected with siKLF7 and with siC, as control. (**d**) Relative cancer stem cell markers expression assessed by RT-qPCR analysis, 72 h post transfection, in OV-90 and PEO1 cells transiently transfected with specific siRNAs (siKLF7) and non-targeting siRNA pool as control (siC). **e** Representative western blot and densitometric analysis and (**f**) immunofluorescence analysis of CD44 expression in OV-90 and in PEO1 cells transiently transfected with siKLF7 and with siC, as control. Cells were harvested 72 h post transfection and cell lysates were subjected to western blot with the specific antibody. GAPDH was used as loading control. For immunofluorescence, the images show the merged signal of Alexa Fluor 488-CD44 mouse monoclonal antibody (green) and DAPI (4′,6-Diamidine-2′-phenylindole) (blue) in siC and siKLF7 OV-90 and PEO1, 72 h post transfection (40x, scale bar 50 μm). For (**a, b, d**) figures, data are presented as fold change, calculated with the ΔΔCt method, using siC as reference sample. Bars and error bars refer to mean and SD (Standard Deviation) of three or more experiments. N.d. = not detectable. To establish statistically significant differences, unpaired t-test was carried out: *p < 0.05, **p < 0.01

In siKLF7 OV-90 cells, 72 h post-transfection we found a marked downregulation in mRNA expression of ZEB2 (*p* = 0.08), Vimentin and MMP2 (*p* < 0.05 for both genes) when compared to scrambled control siRNA (Fig. 4a). These modulations were confirmed at the protein levels for both ZEB2 and Vimentin (Fig. 4a). Likewise, both mRNA and protein expression of SNAIL, ZEB2 and Vimentin were significantly reduced in response to KLF7 knockdown in PEO1 cells (Fig. 4b). Notably, despite the downregulation in SNAIL levels, we did not detect a significant increase in E-cadherin levels in PEO1, probably because it occurs longer after the reduction of SNAIL. Finally, gelatin zymography showed reduced activity of MMP2 in conditioned medium of both siKLF7 OV-90 and PEO1 cells when compared to their control counterparts (Fig. 4c).

### KLF7 knockdown suppresses expression of stem cells markers

There is accumulating evidence for a highly relevant overlap between stemness, cancer, and EMT [50]. Therefore, we sought to verify whether KLF7 can also promote stemness in HGSOC. To this end we employed RT-qPCR analysis, western blot and/or immunofluorescence to assess the expression of CD44, NANOG, OCT4 and SOX2, regarded as putative ovarian cancer stem cells markers [51, 52]. Results obtained showed in OV-90 cells a significant down-regulation of CD44 expression at both the mRNA and protein levels following KLF7 silencing (Fig. 4d, e and f). In PEO1 cells, CD44 was almost undetectable at the western blot analysis, while low levels of staining were found at the immunofluorescence examination and, therefore, no definitive conclusions could be drawn for this cell line (Fig. 4e and f). No other significant changes were observed.

### KLF7 knockdown inhibits spheroid formation of HGSOC cells

**Fig. 5:**
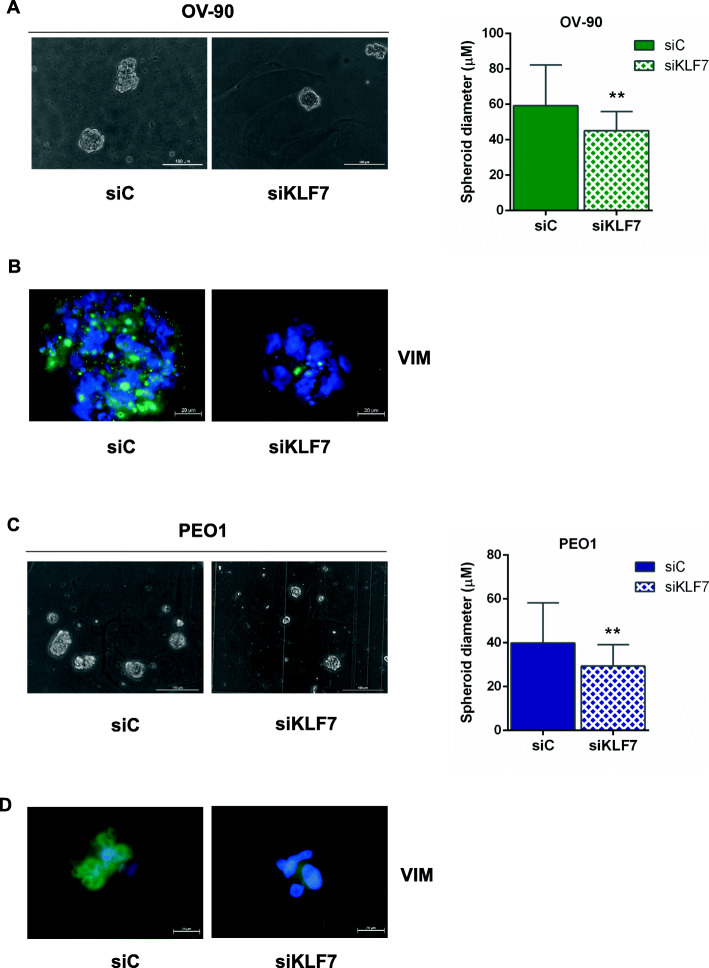
KLF7 silencing reduces HGSOC spheroid size. **a, c** Spheroid assay results using OV-90 and PEO1 cells transfected with siKLF7 and with siC, as control, after 10 days of culture. Left panel: representative bright field microscopy (20x; scale bars, 100 μm) images of transfected OV-90 and PEO1 spheroids released from the hydrogel. Right panel: bar chart showing spheroid size (μm), measured by Leica Application Suite (LAS) analysis. Bars and error bars refer to mean and SD (Standard Deviation) of three experiments. At least 20 spheroids/condition were analysed for each experiment. **b, d** Representative immunofluorescence (100x; scale bars, 20 μm) images of transfected OV-90 and PEO1 spheroids (siC and siKLF7) released from the hydrogel. The images show the merged signal of Alexa Fluor 488-Vimentin (VIM) mouse monoclonal antibody (green) and DAPI (40,6-diamidino-2-phenylindole) (blue). To establish statistically significant differences, unpaired t-test was carried out: **p < 0.01

In keeping with the notion that cancer 3D culture methods better recapitulate in vivo growth conditions, thus allowing more faithful reproduction of broader aspects of tumor biology than conventional 2D monolayer cultures [53], we developed in vitro 3D culture from OV-90 and PEO1 cells transiently silenced for KLF7. PEO1 cells only formed small clusters, with very dense islands of small cells tightly attached. Results obtained showed that KLF7 depletion impaired spheroid growth of OV-90 and PEO1 cells, as demonstrated by reduced spheroid dimensions in siKLF7 compared to siC cells (*p* < 0.01 for both, Fig. 5a and c). In addition, both OV-90 and PEO1-derived spheroids were subjected to immunofluorescence staining for Vimentin expression (Fig. 5b and d). In line with results obtained by western blot analysis on 2D cell cultures, we observed a reduction in Vimentin levels following KLF7 silencing. On the whole, these findings are in line with previous data showing that, in ovarian cancer, the ability to form spheroids correlates with the presence of abundant vimentin [54].

### Structural analysis of the modelled protein and identification of binding sites

**Fig. 6:**
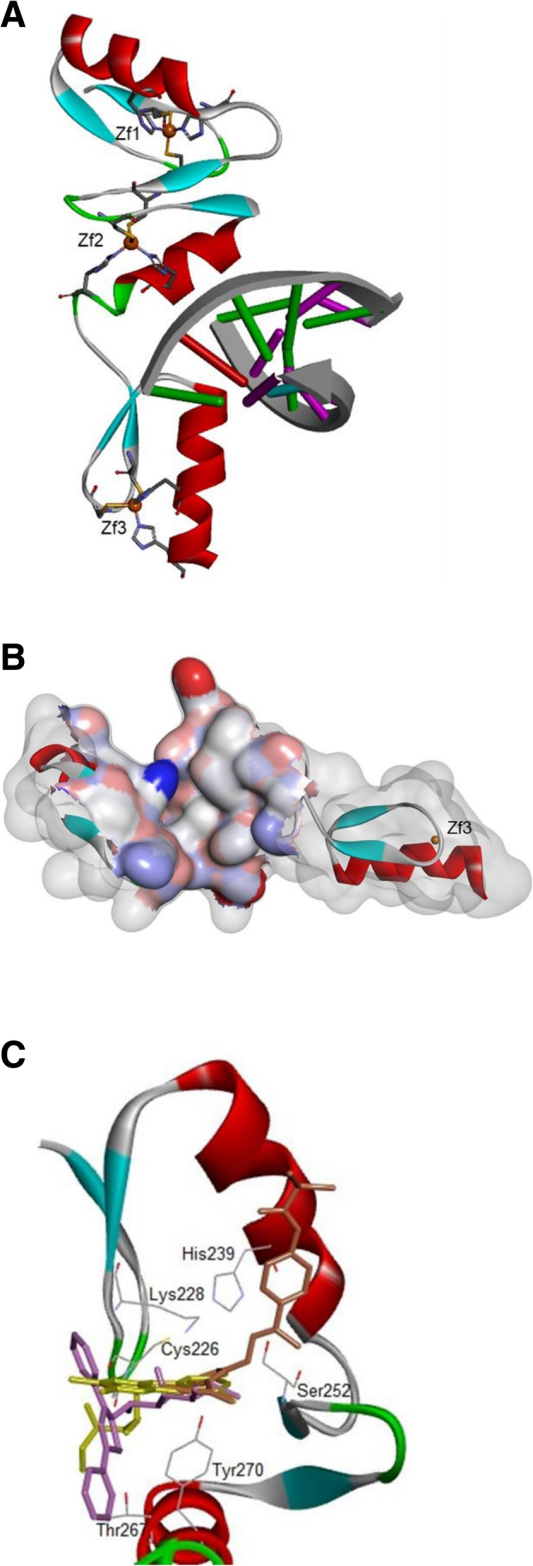
Computational analysis and predictive modelling of discovery of small molecule inhibitors of KLF7-DNA interaction interface. **a** The predicted model structure of the zinc-finger domain of KLF7. The overall fold of KLF7 monomer in complex with DNA is presented as a cartoon model. Zinc ions are shown as orange spheres. **b** Representation of the most preferable druggable site in KLF7. The binding pocket, identified by SiteMap and metaPocket, is shown as solvent surface, coloured by atom charge. **c** Overlay of the top three compounds. Compound#1 (yellow), Compound#2 (violet), Compound#3 (orange). Main interacting residues for the three scaffolds are displayed

The three zinc fingers of KLF7 (residues 218–302) showed 76% sequence identity with residues 399–483 of the crystal structure of the zinc finger domain of KLF4 bound to its target DNA (PDB code: 2WBS). This allowed the zinc finger domain of KLF7 to be modeled confidently by homology modelling using the program Modeller. To validate the computational model multiple approaches were employed. The PROCHECK suite of programs was used to check the stereochemical quality of protein structure and has shown that 90.9% of the residues fall in the most favored region, 9.1% in the additional allowed region and 0.0% in both generously allowed and disallowed regions. For a good quality model, it is expected that the residues located in the most favorable and additional allowed regions should be more than 90%, which is the case of KLF7 model (100%). To check the compatibility of the residues with their environment we used the program VERIFY3D which revealed that 84.7% of the residues averaged 3D-1D score over 0.2, indicative of a correct fold. Prosa was employed to check the energy of residues using the web service ProSA-web. The Z-score calculated by the program was − 4.24, a value within the range of the experimental protein structures of the same size, indicating a good quality model structure. Through this assessment and analysis process, it is concluded that the model generated in the present study is reliable and can be used to characterize KLF7-inhibitor interactions. The model displays three characteristic C2H2-type zinc finger motifs at its C-terminus used by the transcription factor to bind DNA. The overall structure of the KLF7- DNA complex is shown in Fig. 6a. Each zinc-finger contains two antiparallel β strands followed by a α-helix and binds one zinc ion. Zn1 is tetrahedrally coordinated to the side chains of Cys221, Cys226, His243 and His239, Zn2 to the side chains of Cys256, Cys251, His269 and His273, Zn3 to the side chains of Cys281, Cys284, His297 and His301. The generated model structure did not contain any co-crystallized ligand, therefore determination of binding site for virtual screening was done using the tool SiteMap in Maestro (Schrödinger 2017–4). Three pockets were identified with comparable SiteScores: Site1 (0.866), Site2 (0.838), Site3 (0.812) and the amino acid residues lining the identified pockets are reported in Table 4. Notably Site1 (Fig. 6b), which has the highest SiteScore value, was identified by metaPocket server also as the most favorable ligand binding site in KLF7. Analysis of potential ligand binding sites in KLF7 and their druggability were then used to guide the virtual screening experiments.

**Table 4:** Druggable sites identified by SiteMap

	SiteScore	Residues
Site1	0.866	Gly225, Cys226, Arg227, Lys228, Val229, Tyr230, Hys235, Ala238, Hys239, Thr242, Hys243, Pro248, Tyr249, Lys250, Cys251, Ser252, Trp253, Glu254, Gly255, Cys256, Glu257, Trp258, Arg259, Ser263, Asp264, Leu266, Thr267, Tyr270, Arg271, Thr274, Ala276.
Site2	0.838	Trp258, Phe260, Ala261, Glu265, Arg268, Hys269, Arg271, Lys272, Gly275, Ala276, Lys277, Phe288, Ser289, Arg290, Hys293.
Site3	0.812	His219, Arg220, Cys221, Gln222, Phe223, Ser233, Leu236, Lys237, Gln240, Arg241, His243, Thr244

### Computational virtual screening against KLF7

To screen effective inhibitors for KLF7, we used compounds of NCI Diversity Set III molecules and the Maybridge HitFinder collection against the potential druggable pocket Site1. The Glide virtual screening workflow was used and all the molecules were initially screened by SP mode and in the subsequent stage by XP docking. The top ranked 10 ligands on the basis of docking score were selected and Prime was used to estimate the binding free energy of all the ligands towards KLF7 and provide the correct ranking based upon the MM-GBSA energy score. Docking and MM-GBSA score of the top targeted compounds are reported in Table 5. Superimposition of the three top-ranked docking result based upon the MM-GBSA energy score (Compound#1, Compound#3 and Compound#9) is shown in Fig. 6c. These compounds will be subjected to detailed biological investigation in order to determine their inhibitory activity towards KLF7.

**Table 5:** Docking score and estimated binding free energy of the selected hit compounds

	Docking score	MM-GBSA (kcal/mol)
**#1**	−10.737	**−49.30**
#2	−8.459	−23.19
**#3**	−8.066	**−42.47**
#4	−8.044	−32.42
#5	−7.932	−16.37
#6	−7.580	−36.22
#7	−7.499	−24.72
#8	−7.475	−5.06
**#9**	−7.461	**−38.42**
#10	−7.368	−31.69

## Discussion

Here we present results from a bioinformatic meta-analysis of late-stage HGSOC transcriptome data, focused on KLFs. Univariate and multivariate analyses identified KLF2, KLF5 and KLF7 as prognostic factor for overall survival in this cohort.

Results of bioinformatic analysis on KLF2 and KLF5 were actually not in line with previous literature data indicating that KLF2 may act as tumor suppressor in ovarian cancer, while KLF5 may behave as an oncogene [13, 17]. With regard to KLF2, previous studies reported a reduction in transcript levels in a series of 23 serous ovarian tumor specimens when compared to eight normal ovaries, and in vitro results suggested a role for KLF2 as a tumor repressor [13]. However, the overall activity of this transcription factor may be much more complex in patients. Indeed, it is known that KLF2 plays critical roles in the activation of various immune cells [55]. Relevant to our context is the notion that KLF2 is important for Tregs production, immune cells that in malignancies help cancer cells to evade treatment response [55]. Interestingly, high Treg infiltration in ovarian cancer has been associated with a metastatic phenotype [56]. Looking at KLF5, to the best of our knowledge, the only data available refer to in vitro experiments on SKOV3 cells and showed a role for KLF5 in driving stemness and drug resistance in ovarian cancer [17]. However, SKOV3, although commonly used as models for HGSOC, actually do not closely resemble HGSOC [57]. Therefore, the reliability of findings from Dong and colleagues [17] in the context of HGSOC needs to be investigated. Importantly, literature data also suggest context-dependent proliferative or antiproliferative functions for KLF5, even in the same cell types [5, 58].

Bioinformatic analysis highlighted KLF7 as the most significant prognostic gene in HGSOC, among the 17 family members. Besides, preclinical data showed promise for personalized treatment approach and in silico analysis provided reliable information for drug target interaction prediction.

KLF7, known as the ubiquitous Krüppel-like factor, is widely expressed in numerous human tissues at low levels; alternatively spliced products, resulting in multiple transcript variants, have been also identified and these might have distinct regulatory properties [59]. Over the past years, the protein has been mainly investigated for its role in neuronal morphogenesis and in the pathogenesis of type 2 diabetes [60, 61]. More recently, however, new evidence have become available suggesting a role for KLF7 in cancer development and progression. Specifically, the protein has been identified as an independent predictor of poor outcome in paediatric acute lymphoblastic leukaemia, lung and gastric cancer [12, 62, 63]. Accordingly, functional in vitro experiments demonstrated that KLF7 promotes proliferation, migration and invasion in different cancer cell lines [12, 63–65]. Data from the present study are in line with these previously published findings supporting a uniform oncogenic role for KLF7. However, while other members in the family have been broadly characterized for their functions in various cancer-relevant processes, mechanistic insights relative to KLF7 role in cancer pathogenesis are very limited [9, 66]. Relevant to this aspect, our data showed that, in HGSOC cells, KLF7 plays a prominent role in inducing EMT. Indeed, we have demonstrated, in two different cellular models, that gene silencing correlates with a significant decrease in Vimentin expression. Vimentin is a well-known EMT actor, promoting migration and invasion of different cell types, through different mechanisms of action [67, 68]. Notably, the concomitant decrease in SNAIL and/or ZEB2 expression (two factors implicated in the regulation of Vimentin expression [68]) after KLF7 silencing, actually corroborated our results. More importantly, the role of KLF7 in driving EMT by regulating Vimentin levels, was also confirmed in the spheroid models, these latter more closely resembling the in vivo tumour growth situation. Results from the present study also demonstrated that KLF7 silencing reduces expression and activity of MMP2, a gelatinase shown to be secreted and activated in ovarian cancer and closely correlated with invasion and metastasis of cancer cells and poor survival [69].

Our data also showed in OV-90 cells a reduced expression of CD44, a putative ovarian cancer stem cells markers, this suggesting a possible role for KFL7 in promoting HGSOC progression also by enhancing cancer stem cell properties. Relevant to this issue, recent data not only support evidence of the co-expression of stemness genes and EMT genes in ovarian cancer, but also that EMT activators can actually induce both EMT and stemness properties, suggesting an intriguing model where EMT arises from ovarian cancer stem cells under the right microenvironment and perhaps vice versa [52]. In line with our results, KLF7 has been recently identified as a functional regulator of human pluripotency, promoting hPSC (human pluripotent stem cells) self-renewal [70].

Notably, preliminary analysis using the MatInspector software (Genomatix Software GmbH) [71] allowed us to identify the presence of putative KLF7 binding site in the SNAIL-, ZEB2-, Vimentin- and CD44-promoter regions (data not shown). These results support the hypothesis that KLF7 may regulate these targets at the transcriptional level, although this assumption requires experimental validation.

Overall, the available literature and our data support the idea that KLF7 oncogenic activity may progress via different pathways, mainly affecting the EMT-program and cancer stemness. Therefore, targeting KLF7 by small compounds may open new possibilities for ovarian cancer treatment. Up to now experimental three-dimensional structural information is not available for KLF7, therefore in the current study we firstly generated a computational model structure of the transcription factor that could serve as starting point for structure-based drug design. In silico homology modelling in fact provides a valuable alternative to generate reasonable three-dimensional model structures for drug discovery [72]. Thereafter, based on the characteristics of the target druggable site we used a docking-based virtual screening to identify putative KLF7 inhibitors. Molecular docking is indeed a powerful tool for identifying the binding modes of ligands to receptors of biomedical relevance [73, 74]. However, docking scoring functions employed in high-throughput virtual screening lack accuracy in the approximation of binding affinities of protein-ligand complexes [75]. Hence we filtered the 10 top ranking poses from docking results on the basis of MMG-BSA method, which generally outperforms the scoring functions of docking algorithms [76]. Results obtained have finally allowed the identification of initial hit compounds for further medicinal chemistry optimization to improve their potency and/or selectivity as potential candidates for clinical therapy. Besides, future studies are already planned in our Department to test the efficacy of the virtual hits in preclinical experimental models.

## Conclusion

Collectively, our findings demonstrated for the first time the biological function and underlying mechanism of KLF7 in HGSOC, suggesting it as a promising prognostic marker and therapeutic target. Besides, computational analysis and predictive modelling of discovery have allowed the identification of hit compounds that could be exploited for developing future therapies.

## Supplementary Information

**Additional file 1:** **Table S1A.** Sequences information related to KLF7 siRNA pool. **Table S1B.** KLF7 transcript variants recognized by KLF7 RT-qPCR oligos. **Table S2.** Primer sequences used for RT-qPCR.

**Additional file 2. **Bioinformatic meta-analysis.

## Data Availability

The datasets analysed during the current study are available in the Array Express repository (https://www.ebi.ac.uk/arrayexpress/experiments/E-MTAB-386/), GEO repository (https://www.ncbi.nlm.nih.gov/geo/) and TCGA repository (https://portal.gdc.cancer.gov/).

## Abbreviations

ATCC: American Type Culture Collection
BLAST: Basic Local Alignment Search Tool
BRCA: Breast cancer type 1 and 2 susceptibility protein (BRCA1 and BRCA2)
CD44: CD44 antigen
CDH1/E-CAD: E-cadherin
CI: Confidence Interval
Ct: Cycle threshold
DAPI: 4′,6-Diamidine-2′-phenylindole
DNA: Deoxyribonucleic acid
ECACC: European Collection of Authenticated Cell Cultures
EMT: Epithelial–Mesenchymal Transition
F test: Fisher F test
FBS: Foetal Bovine Serum
FIGO stage: Fédération Internationale de Gynécologie et d’Obstétrique staging system
GAPDH: Glyceraldehyde-3-phosphate dehydrogenase
GEO: Gene Expression Omnibus
GSE: Gene Expression Omnibus Series
HGSOC: High-Grade Serous Ovarian Cancer
hPSC: Human pluripotent stem cells
HR: Hazard Ratio
KLFs: Kruppel-like factors
LAS: Leica Application Suite
MEM: Minimum Essential Medium
MM-GBSA: Molecular Mechanics Generalized Born Surface Area
MMP2: Matrix Metalloproteinase 2
MMP9: Matrix Metalloproteinase 9
mRNA: Messanger ribonucleic acid
NANOG: Homeobox NANOG
NCI: National Cancer Institute
OCT4: Octamer-binding transcription factor 4
OS: Overall Survival
P: *p*-value
PAINS: pan-assay interference compounds
PARP: Poly (ADP-ribose) polymerase
PBS: Phosphate-buffered saline
PDB: Protein data bank
PDF: probability density function
RNA: Ribonucleic acid
RNA-seq: RNA-sequencing
RPLP0: Ribosomal Protein Lateral Stalk Subunit P0
RPMI: Roswell Park Memorial Institute Medium
RT-qPCR: Real Time Quantitative Polymerase Chain Reaction
SDS-PAGE: Sodium Dodecyl Sulphate - PolyAcrylamide Gel Electrophoresis
SD: Standard Deviation
siC: siRNA control
iKLF7: siRNA targeted to KLF7
siRNA: Small Interfering RNA (o short interfering RNA)
SLUG: Snail Family Transcriptional Repressor 2 (SNAI2)
SNAIL: Snail Family Transcriptional Repressor 1 (SNAI1)
SNRPD3: Small Nuclear Ribonucleoprotein D3 Polypeptide
SOX2: SRY (sex determining region Y)-box transcription factor 2
SP: Standard Precision
STR: Short Tandem Repeat
TCGA-OV: The Cancer Genome Atlas Ovarian Cancer
TFs: Transcription Factors
TWIST 1: Twist family basic helix-loop-helix (bHLH) transcription factor 1
TWIST 2: Twist family basic helix-loop-helix (bHLH) transcription factor 2
VIM: Vimentin
XP: Extra precision
ZEB1: Zinc Finger E-Box Binding Homeobox 1
ZEB2: Zinc Finger E-Box Binding Homeobox 2
