# Supplementary material for: KLF7: a new candidate biomarker and therapeutic target for high-grade serous ovarian cancer
Source: J Exp Clin Cancer Res. 2020 Nov 30;39:265. doi: 10.1186/s13046-020-01775-9 (PMC7702713; doi:10.1186/s13046-020-01775-9)
Supplement: Supplementary file 1 — Additional file 1: Table S1A. Sequences information related to KLF7 siRNA pool. Table S1B. KLF7 transcript variants recognized by KLF7 RT-qPCR oligos. Table S2. Primer sequences used for RT-qPCR. [file 13046_2020_1775_MOESM1_ESM.docx]

**Supplementary File 1**

**KLF7: A NEW CANDIDATE BIOMARKER AND THERAPEUTIC TARGET FOR HIGH-GRADE SEROUS OVARIAN CANCER**

De Donato *et al*.

This Supplementary Material includes:

- Table S1
- Table S2

| **SMART POOL ON-TARGET PLUS KLF7** | **Sequence** | **KLF7 transcript variant** | **Targeting sequence position (bp)** |
| --- | --- | --- | --- |
| **siRNA #1** | GUUCACCGCUGUCAGUUUA | NM_003709 | 1032 -1050 |
|  |  | NM_001270943 | 657-675 |
|  |  | NM_001270944 | 621-639 |
| **siRNA #2** | GGGACAAGUUGCUAUCUGA | NM_003709 | 682-700 |
|  |  | NM_001270942 | 540-558 |
|  |  | NM_001270943 | 307-325 |
|  |  | NM_001270944 | 271-289 |
| **siRNA #3** | CAGCAGACAUGCCUUGAAU | NM_003709 | 477-495 |
|  |  | NM_001270942 | 335-353 |
| **siRNA #4** | GCUCUUCUCUAGACAGCUA | NM_003709 | 727-745 |
|  |  | NM_001270942 | 585-603 |
|  |  | NM_001270943 | 352-370 |
|  |  | NM_001270944 | 316-334 |

**Table S1A.** Sequences information related to KLF7 siRNA pool.

**Table S1B.** KLF7 transcript variants recognized by KLF7 RT-qPCR oligos.

| **RT-qPCR oligos** | **Sequence** | **KLF7 transcript variant** | **Amplicon position (bp)** |
| --- | --- | --- | --- |
| **KLF7** | Fw:ACTGTTTCCTCCACGCTTCCC  Rv: GTCCCGAGAGAGCAAGATGTCC | NM_003709 | 556-686 |
|  |  | NM_001270942 | 414-544 |
|  |  | NM_001270943 | 181 -311 |
|  |  | NM_001270944 | 145 -275 |

**Table S2.** Primer sequences used for RT-qPCR.

| **GENE ID** | **Gene Symbol** | **Primer Forward** | **Primer Reverse** | **Amplicon Length (bp)** |
| --- | --- | --- | --- | --- |
| 960 | CD44 | GGGCTGGGCTTAGACAGAGTTG | GGAGAGGGTAGACAGGGAGGAG | 102 |
| 999 | E-CAD | CCAACAAAGACAAAGAAGGCAAGG | AGTGTATGTGGCAATGCGTTCTC | 149 |
| 8609 | KLF7 | ACTGTTTCCTCCACGCTTCCC | GTCCCGAGAGAGCAAGATGTCC | 131 |
| 4313 | MMP2 | CTCCAATCCCACCAACCCTC | CCAGTGCCCTCTTGAGACAG | 174 |
| 4318 | MMP9 | GGGGAAGATGCTGCTGTTCAG | CGTCGTGCGTGTCCAAAGG | 128 |
| 79923 | NANOG | AATACCTCAGCCTCCAGCAGATG | TGCGTCACACCATTGCTATTCTTC | 148 |
| 5460 | OCT4 | AGAGGATCACCCTGGGATATACAC | GCCGCAGCTTACACATGTTCTTG | 138 |
| 6175 | RPLP0 | GCGACCTGGAAGTCCAAC | CACATTGTCTGCTCCCACAA | 87 |
| 6591 | SLUG | GGCAAGATGCCGCGCTCCTT | GCCATTGGGTAGCTGGGCGT | 222 |
| 6615 | SNAIL | GTCAGGAAGCCCTCCGACCCCAATC | CCGAAGGGAGGCCCAGGCAAT | 216 |
| 6634 | SNRPD3 | GCTCATTGAAGCAGAGGACAA | CCACTCGGCCATCTCTGTA | 74 |
| 6657 | SOX2 | AGGGGGAAAGTAGTTTGCTGCCTCT | TGCCGCCGCCGATGATTGTT | 136 |
| 7291 | TWIST1 | TAAAAGTGCGCCCCACGCCC | CAGCAGGGCCGGAGACCTAGA | 169 |
| 117581 | TWIST2 | CGCGCCAGGAGGAGATTCTGAAT | ATGTGCTCACTCCCGCCAACG | 127 |
| 7431 | VIM | CACCAGCCGCAGCCTCTAC | CGAGAAGTCCACCGAGTCCTG | 121 |
| 6935 | ZEB1 | GGAGGAGGAGGAAGAAGTGGAAG | ACTTGCTCACTACTCTCGCCTAC | 150 |
| 9839 | ZEB2 | ATGACCTGCCACCTGGAACTC | GCGGTACTTGATGTGCTCCTTC | 110 |
