# Supplementary material for: KLF7: a new candidate biomarker and therapeutic target for high-grade serous ovarian cancer
Source: J Exp Clin Cancer Res. 2020 Nov 30;39:265. doi: 10.1186/s13046-020-01775-9 (PMC7702713; doi:10.1186/s13046-020-01775-9)
Supplement: Supplementary file 2 — Additional file 2. Bioinformatic meta-analysis. [file 13046_2020_1775_MOESM2_ESM.pdf]

Supplementary File 2

KLF7: A NEW CANDIDATE BIOMARKER AND THERAPEUTIC TARGET FOR HIGH-GRADE SEROUS OVARIAN CANCER

De Donato *et al*.

This supplementary material includes

- Curated Ovarian Cancer datasets - KLF family

KLF1

Univariate

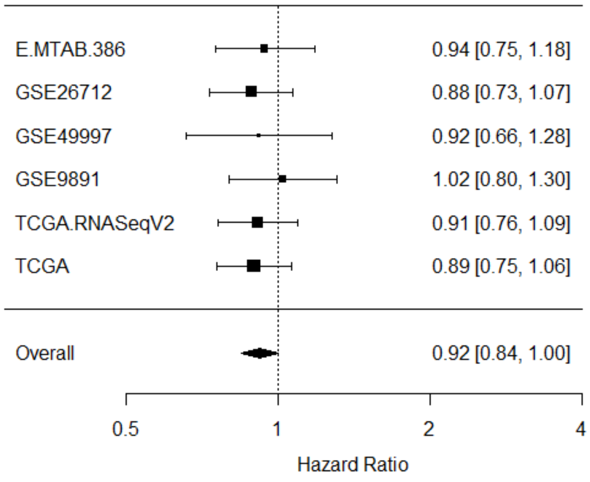

Pval: 0.04780519

Multivariate
(age, FIGO stage, residual tumour)

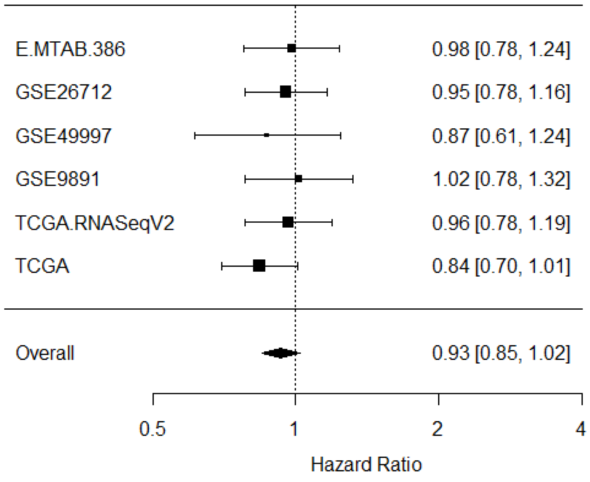

Pval: 0.1240095

KLF2

Univariate

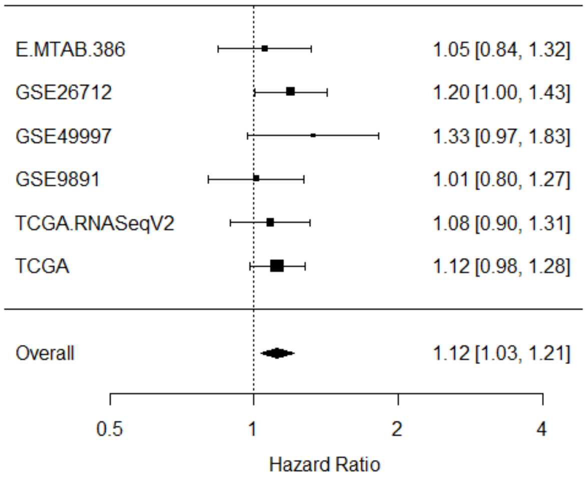

Pval: 0.004967524

Multivariate
(age, FIGO stage, residual tumour)

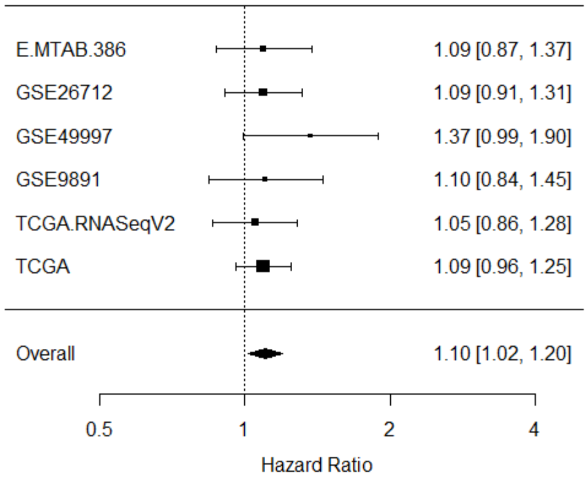

Pval: 0.01909106

KLF3

Univariate

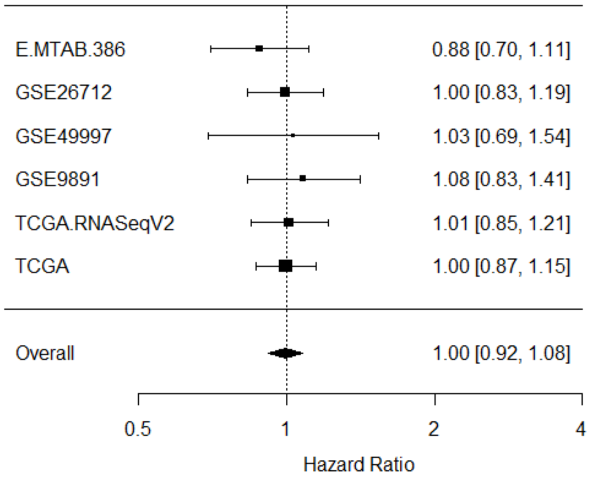

Pval: 0.9180043

Multivariate
(age, FIGO stage, residual tumour)

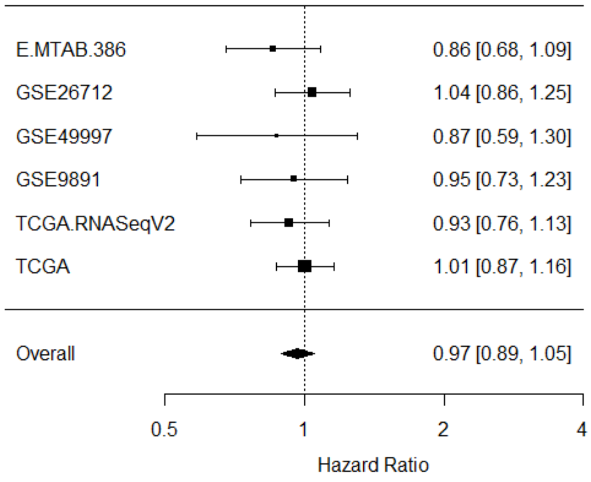

Pval: 0.4121924

KLF4

Univariate

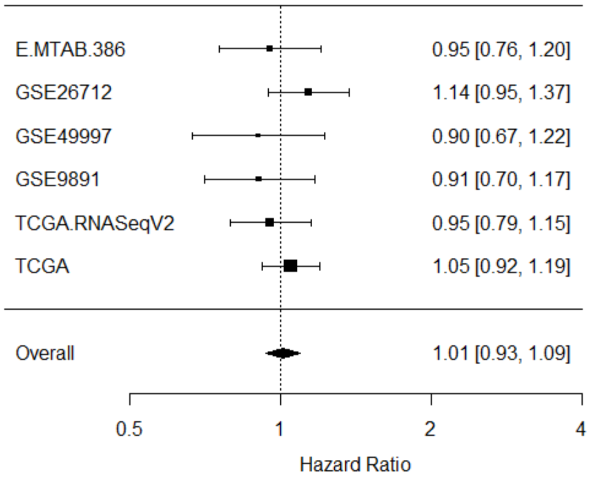

Pval: 0.8157031

Multivariate
(age, FIGO stage, residual tumour)

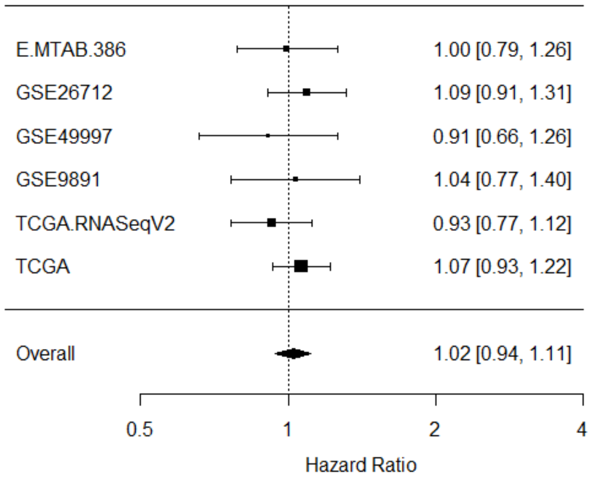

Pval: 0.5794065

KLF5

Univariate

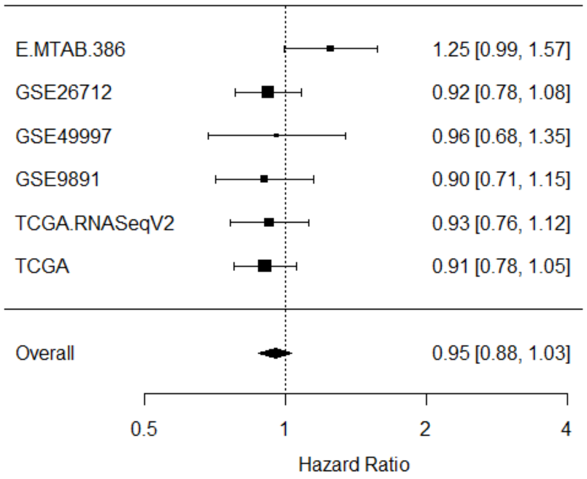

Pval: 0.2454189

Multivariate
(age, FIGO stage, residual tumour)

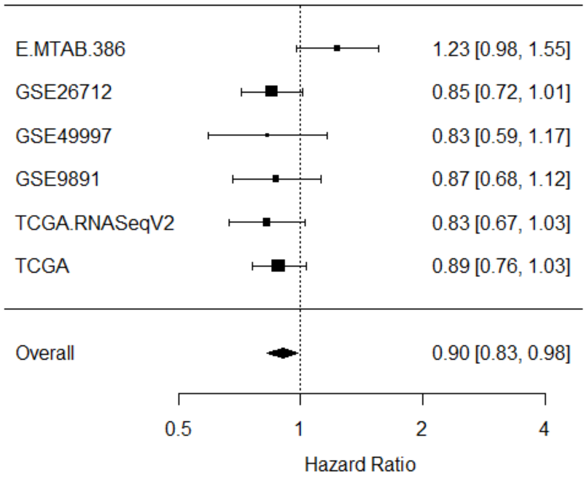

Pval: 0.01732922

KLF6

Univariate

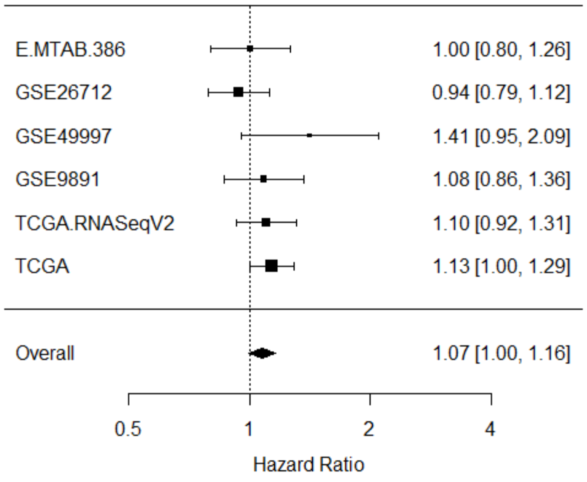

Pval: 0.06464532

Multivariate
(age, FIGO stage, residual tumour)

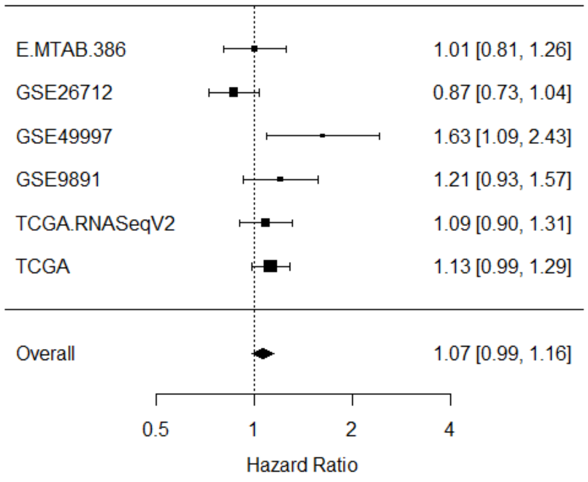

Pval: 0.09921779

KLF7

Univariate

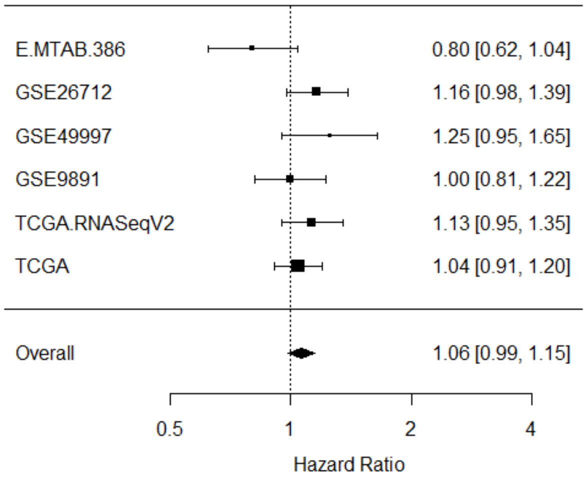

Multivariate
(age, FIGO stage, residual tumour)

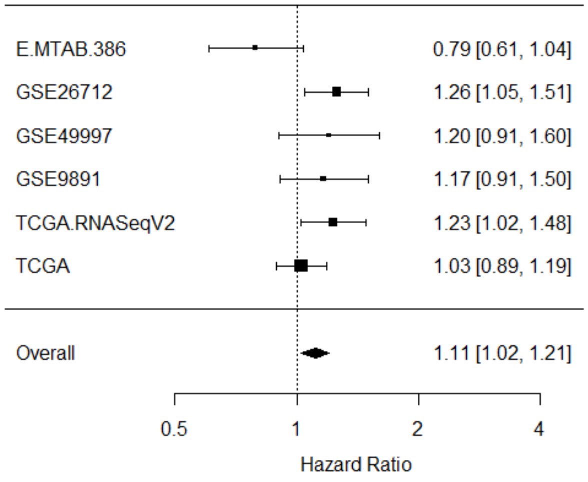

Pval: 0.1079501

Pval: 0.01172062

KLF8

Univariate

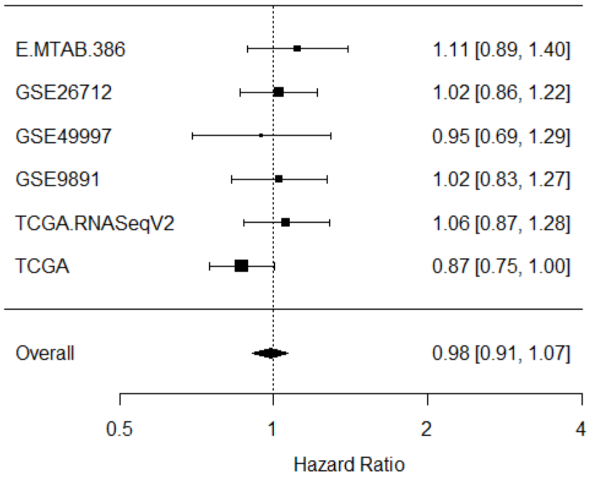

Pval: 0.6953746

Multivariate
(age, FIGO stage, residual tumour)

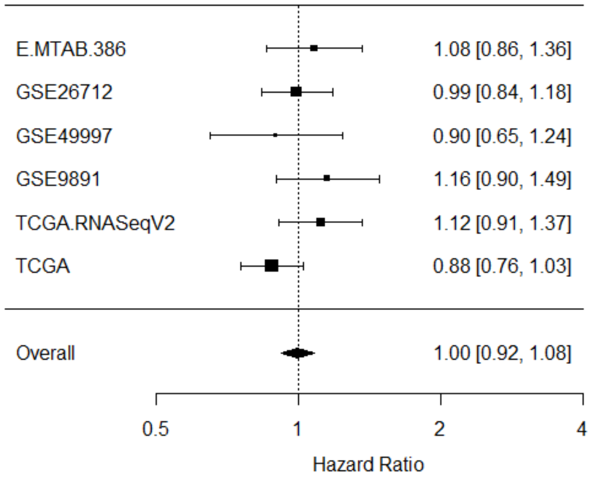

Pval: 0.9572073

KLF9

Univariate

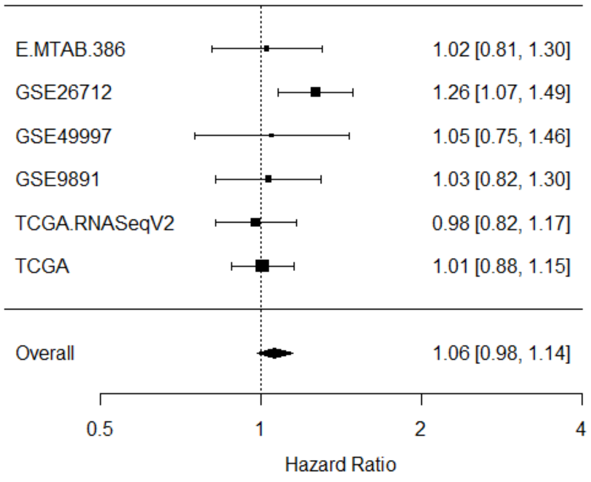

Pval: 0.1416893

Multivariate
(age, FIGO stage, residual tumour)

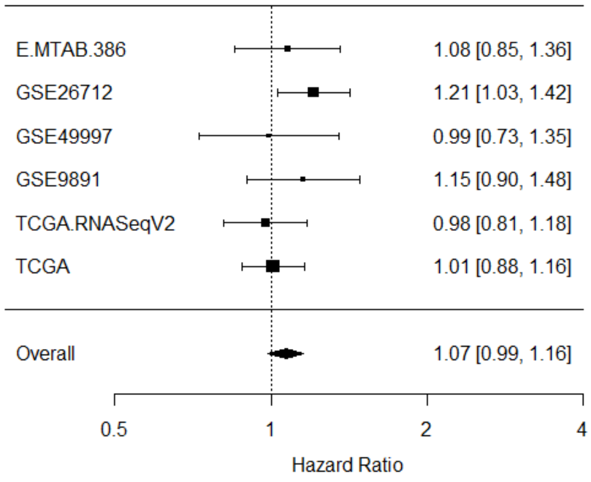

Pval: 0.09454739

KLF10

Univariate

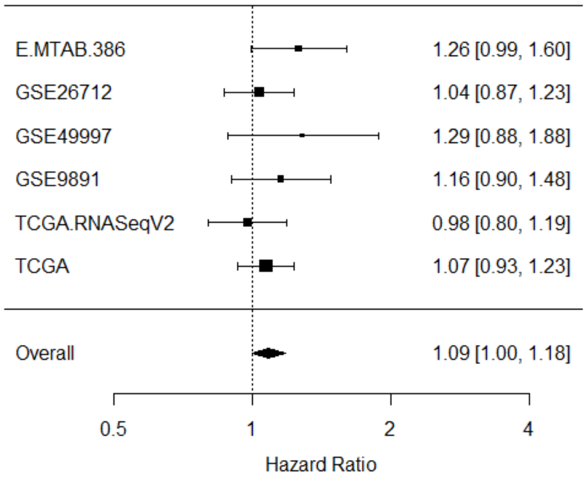

Pval: 0.05060688

Multivariate
(age, FIGO stage, residual tumour)

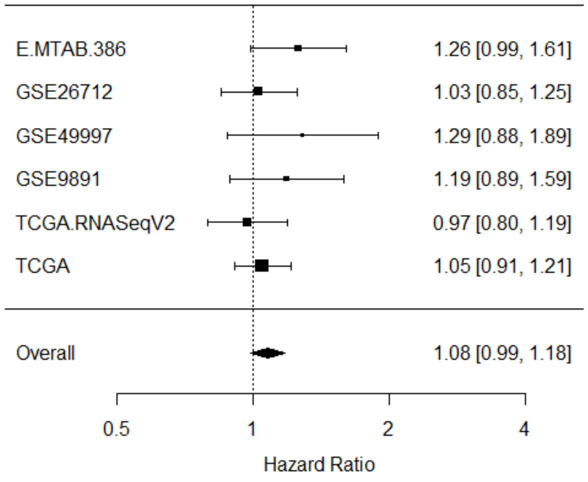

Pval: 0.08150034

KLF11

Univariate

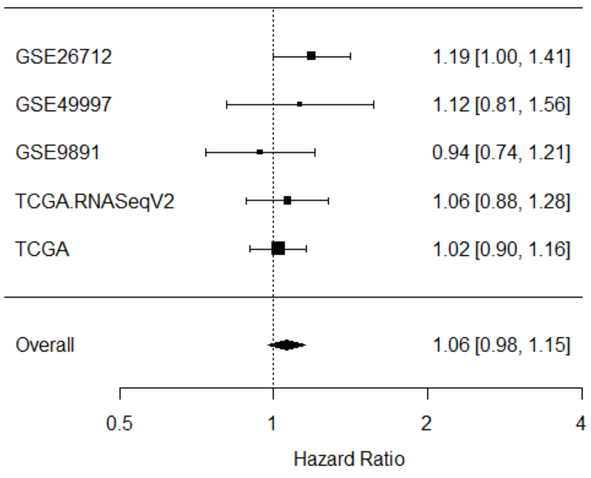

Pval: 0.1593252

Multivariate
(age, FIGO stage, residual tumour)

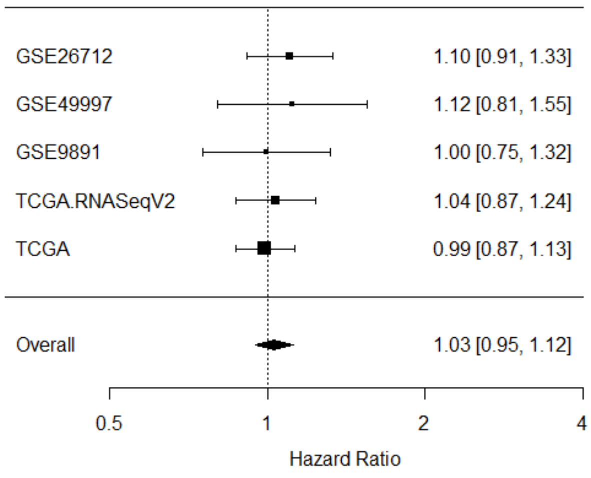

Pval: 0.466779

KLF12

Univariate

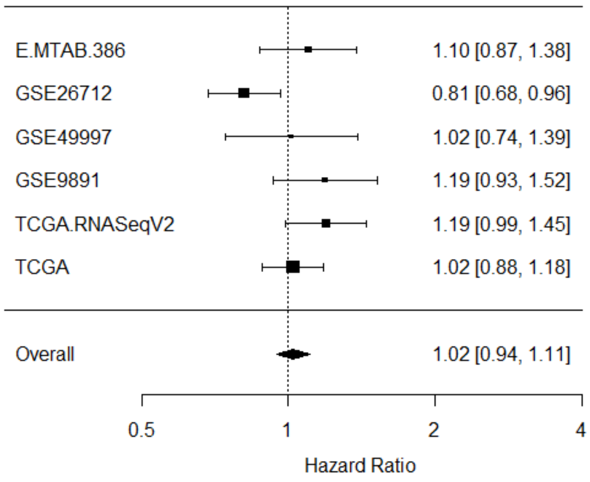

Pval: 0.5730336

Multivariate
(age, FIGO stage, residual tumour)

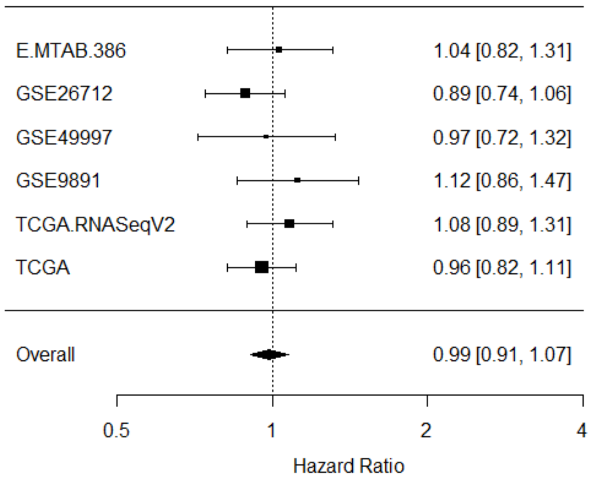

Pval: 0.7917701

KLF13

Univariate

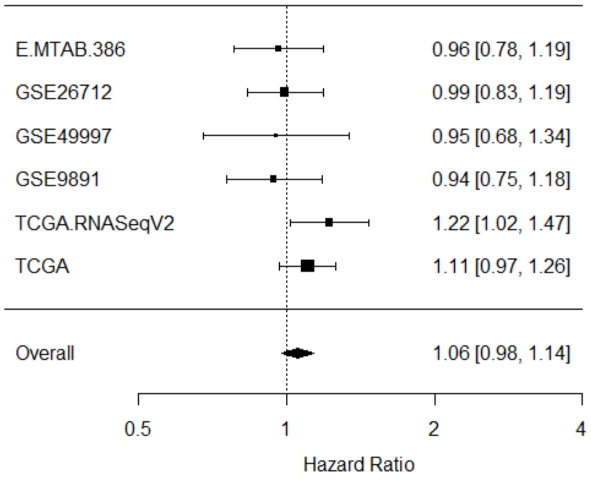

Pval: 0.1708605

Multivariate
(age, FIGO stage, residual tumour)

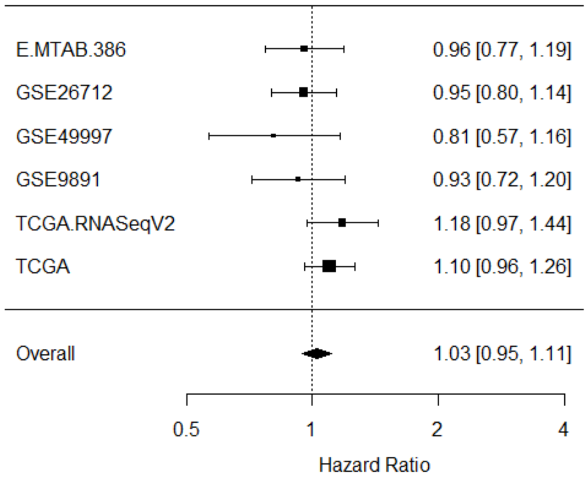

Pval: 0.5229142

KLF14

Univariate

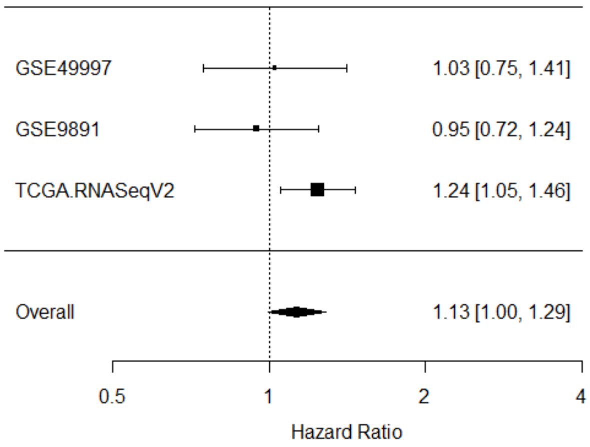

Pval: 0.05937618

Multivariate
(age, FIGO stage, residual tumour)

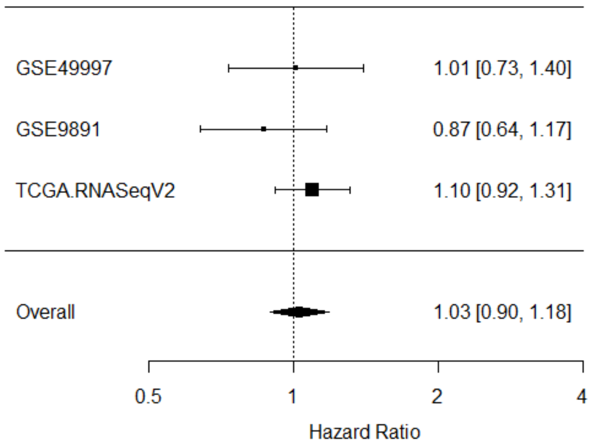

Pval: 0.6873131

KLF15

Univariate

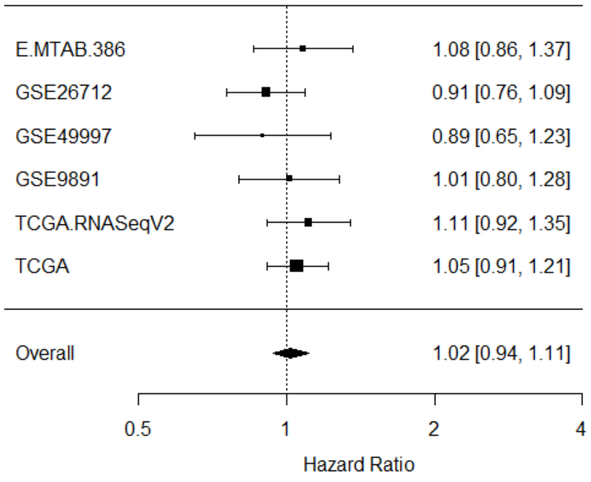

Pval: 0.6119663

Multivariate
(age, FIGO stage, residual tumour)

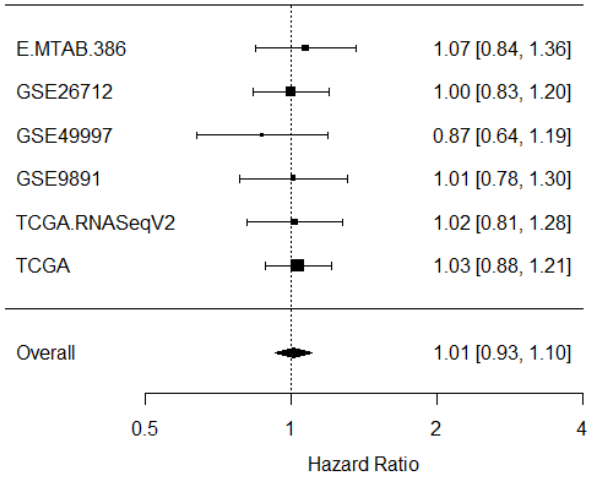

Pval: 0.8062681

KLF16

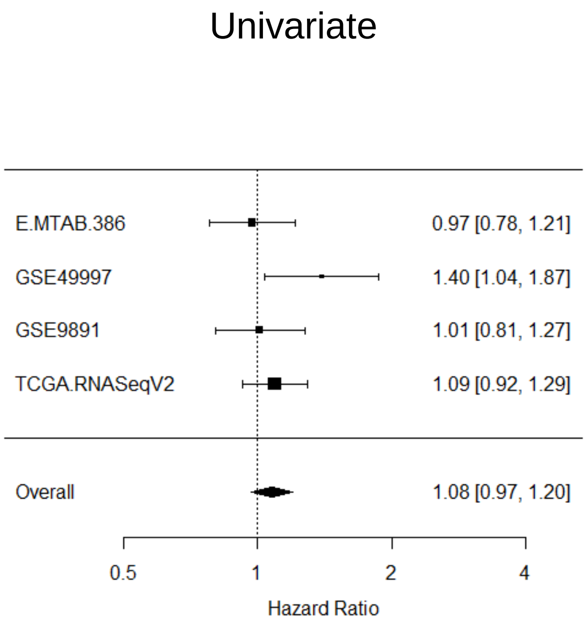

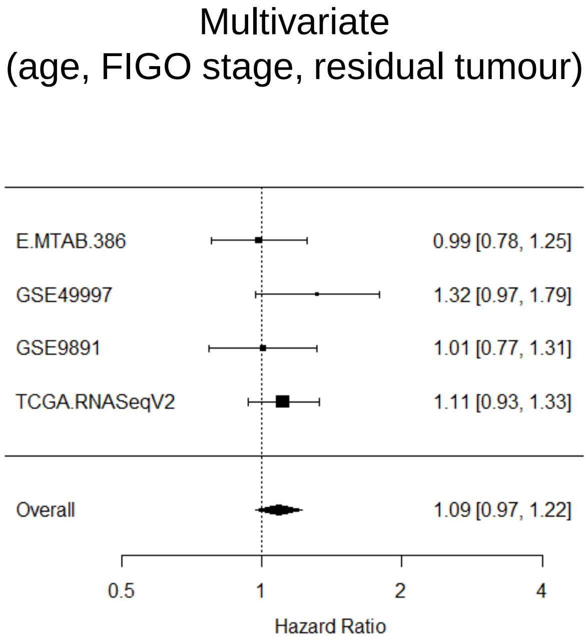

Pval: 0.164804

Pval: 0.1576571

KLF17

Univariate

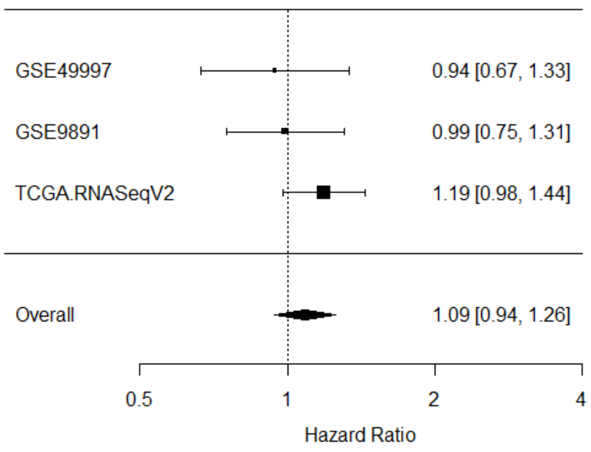

Pval: 0.2474039

Multivariate
(age, FIGO stage, residual tumour)

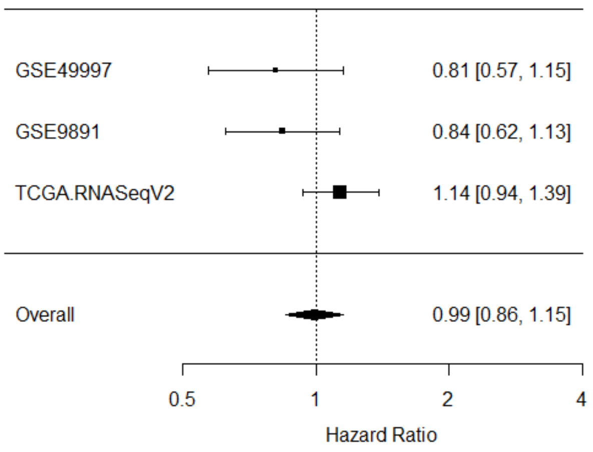

Pval: 0.9410717
